# A multi‐stage 3D convolutional neural network algorithm for CT‐based lung segment parcellation

**DOI:** 10.1002/acm2.70193

**Published:** 2025-07-23

**Authors:** Trishul Siddharthan, Zhoubing Xu, Bruce Spottiswoode, Chris Schettino, Yoel Siegel, Michalis Georgiou, Thomas Eluvathingal, Bernhard Geiger, Sasa Grbic, Partha Gosh, Rachid Fahmi, Naresh Punjabi

**Affiliations:** ^1^ Division of Pulmonary, Critical Care and Sleep Medicine University of Miami Miami Florida USA; ^2^ Siemens Medical Solutions USA, Inc. Malvern Pennsylvania USA; ^3^ Department of Radiology Miller School of Medicine University of Miami Miami Florida USA; ^4^ Department of Nuclear Medicine Miller School of Medicine University of Miami Miami Florida USA

**Keywords:** airways disease, CT‐based lung segment parcellation, deep learning

## Abstract

**Background:**

Current approaches to lung parcellation utilize established fissures between lobes to provide estimates of lobar volume. However, deep learning segment parcellation provides the ability to better assess regional heterogeneity in ventilation and perfusion.

**Purpose:**

We aimed to validate and demonstrate the clinical applicability of CT‐based lung segment parcellation using deep learning on a clinical cohort with mixed airways disease.

**Methods:**

Using a 3D convolutional neural network, airway centerlines were determined using an image‐to‐image network. Tertiary bronchi were identified on top of the airway centerline, and the pulmonary segments were parcellated based on the spatial relationship with tertiary and subsequent bronchi. The data obtained by following this workflow was used to train a neural network to enable end‐to‐end lung segment parcellation directly from 123 chest CT images. The performance of the parcellation network was then evaluated quantitatively using expert‐defined reference masks on 20 distinct CTs from the training set, where the Dice score and inclusion rate (i.e., percentage of the detected bronchi covered by the correct segment) between the manual segmentation and automatic parcellation results were calculated for each lung segment. Lastly, a qualitative evaluation of external validation was performed on 20 CTs prospectively collected by having two radiologists review the parcellation accuracy in healthy individuals (*n* = 10) and in patients with chronic obstructive pulmonary disease (COPD) (*n* = 10).

**Results:**

Means and standard deviation of Dice score and inclusion rate between automatic and manual segmentation of twenty patient CTs were 86.81 (SD = 24.54) and 0.75 (SD = 0.19), respectively, across all lung segments. The mean age of the qualitative dataset was 54.4 years (SD = 16.4 years), with 45% (*n* = 9) women. There was 99.2% intra‐reader agreement on average with the produced segments. Individuals with COPD had greater mismatch compared to healthy controls.

**Conclusions:**

A deep‐learning algorithm can create parcellation masks from chest CT scans, and the quantitative and qualitative evaluations yielded encouraging results for the potential clinical usage of lung analysis at the pulmonary segment level among those with structural airway disease.

## 1 INTRODUCTION

Computed tomography (CT) is part of the standard assessment of a range of respiratory conditions, and when combined with single‐photon or positron‐emission tomography, it can provide assessments of regional ventilation, perfusion, and metabolic activity.^1^ Heterogeneity in lung function is a hallmark of acute and chronic respiratory disease.^2^ Diseases, such as COPD and interstitial lung disease, have geospatial and temporal patterns of injury that result in unique ventilation and perfusion profiles.^2^ Ventilation and perfusion heterogeneity from lung disease can be a marker of unique disease trajectories and have therapeutic implications.^3^ Lung volume reduction, a technique used for COPD management, aims to improve overall ventilation heterogeneity by selectively removing sections of the ventilated lung.^4^ Furthermore, delivery of inhaled medications for respiratory disease may preferentially deposit in areas of higher ventilation.^5^

To date, assessment of regional heterogeneity in ventilation and perfusion has largely been at the lobar level, utilizing fissures to define anatomic landmarks to define differences between lobes to guide surgical resection. Automated CT‐based parcellation algorithms for the lung lobes are now commonplace, but the ability to robustly delineate lung segments remains a challenge.^6^ Novel techniques to generate segmental masks can pave the way to improve precision therapies, as well as better characterize heterogeneity of lung disease. Deep‐learning algorithms have the potential to generate standardized segmentation masks by determining airway centerlines and parcellating segments based on spatial relationships with tertiary bronchi, thereby allowing for assessment of ventilation and perfusion heterogeneity between segments of the lung.^7^

**FIGURE 1:**
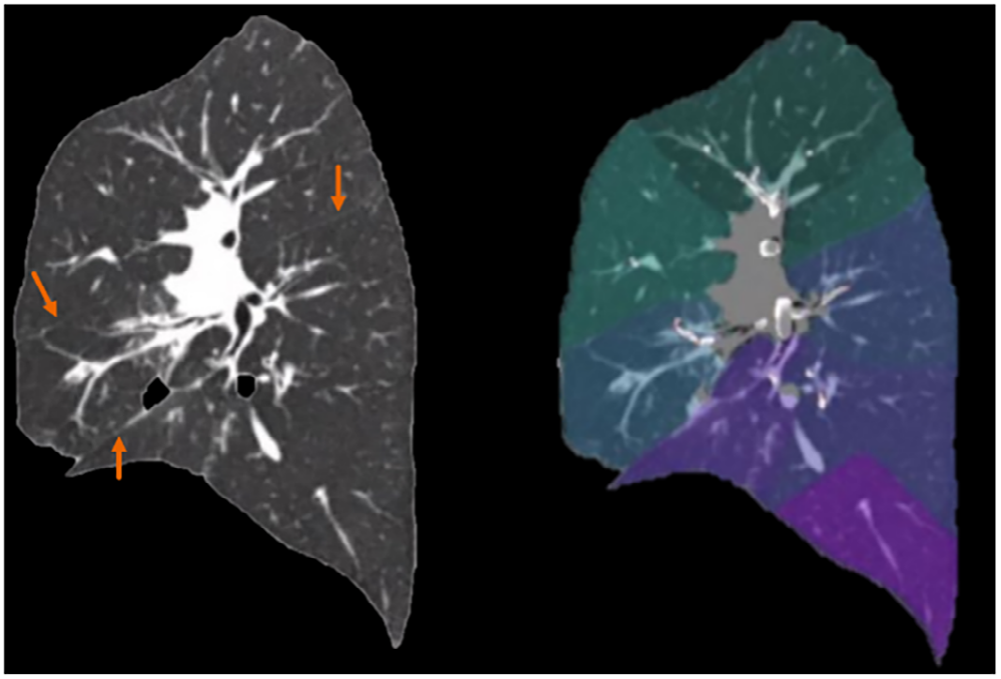
Lung Parcellation using Deep Image‐to‐Image Network. A sample case showing the challenging task of lung segment parcellation directly from CT data. Fissures are noted with orange arrows. There are no clear visible boundaries between most adjacent segments, and meticulous navigation on the CT is required while evaluating bronchi and pulmonary vessel pathways. Left: CT slice with visible lung fissures shown in orange arrows. Right: automatic segment parcellation results on the same CT slice. CT, computed tomography

The pulmonary segment refers to a section of the lung that is supplied by its own bronchus and artery. Each segment is anatomically and functionally distinct, which means that one segment can be surgically removed without affecting the other segments. These segments are also considered subdivisions of lung lobes, with a total of 18 segments (with some left segments fused together by convention).^8^, ^9^ On clinically acquired CT images, the anatomical boundaries between most pulmonary segments are not readily visible (Figure 1).

This poses a challenge not only in preparing annotations for machine‐learning algorithms but also in validating the algorithms’ outcomes. In other words, there is no easy‐to‐access gold standard, unless through open surgery or a well‐crafted phantom.

There is limited published literature on this topic given these challenges. Busayarat and colleagues proposed an interactive approach, where the pulmonary segments were computed with region growing from the manually annotated tertiary bronchi.^10^ Rikxoort et al. presented an automatic approach for pulmonary segment parcellation by training a classifier on relative locational features of the segments. Although these earlier attempts had limited degrees of automation and accuracy, there have been no approaches described to date that derive pulmonary segments based on bronchial segments. Automated parcellation techniques have previously been compared in other organ systems without defined boundaries, with various advantages and disadvantages; however, these approaches do not exist for lung parcellation.^11^

An automated lung segment parcellation algorithm has the potential to be used widely in clinical practice; however, no studies to date have validated this approach in a clinical cohort with structural airway disease. We aimed to develop a deep learning CT‐based lung segment parcellation algorithm and validate parcellation results among adults with and without lung disease.

## 2 METHODS

### 2.1 Lung segment parcellation algorithm

To generate ground truth labels for training, we extract the airway trees, label the segment bronchi from the airway tree, then subdivide the lung lobe into lung segments using a derivation dataset. A convolutional neural network (CNN) was then trained to parcellate pulmonary segments from input CT images. Several components in the proposed approach, including lung lobe segmentation and airway tree tracing, are based on prior work.^12^, ^13^

### 2.2 Data preparation

**FIGURE 2:**
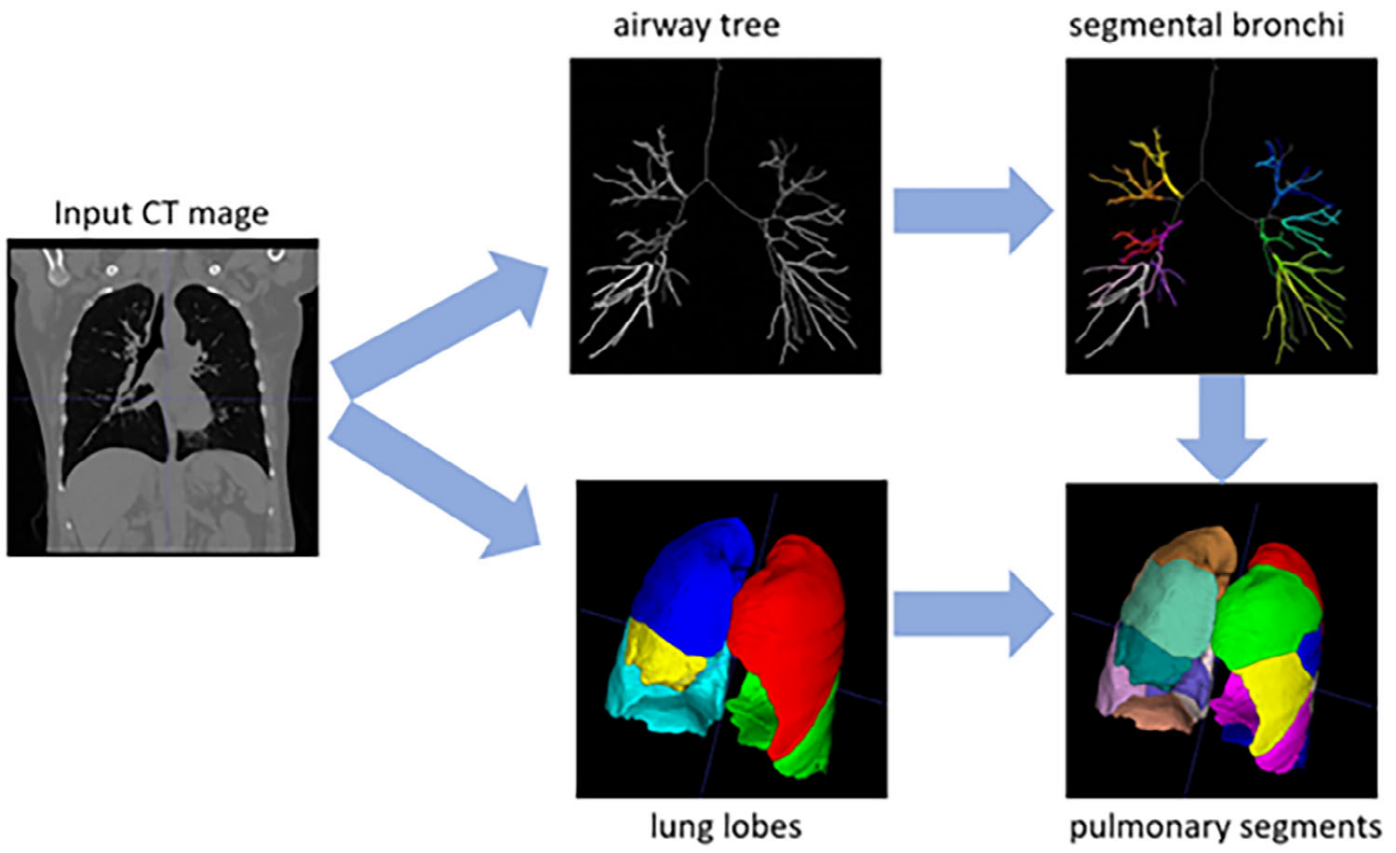
Pulmonary segment annotation workflow. This included airway tree tracing, labeling of tertiary (and subsequent) bronchi, and segmental parcellation based on proximity to the airway tree labels and respecting lobe boundaries from an existing lung lobe algorithm.

The workflow to generate ground truth training labels is summarized in Figure 2.

#### 2.2.1 Lung lobe parcellation

An image‐to‐image CNN was employed to generate lung lobe parcellation based on an input CT image.^12^ To address abnormal lung parcellation, particularly in cases involving pneumonia patterns, the algorithm is fine‐tuned on cases with abnormality patterns, and the network architecture is adjusted to regularize the lung shape. This ensures that higher intensity pneumonia regions (if present) are not overlooked and are appropriately captured in the parcellation outcome.

#### 2.2.2 Airway tree tracing

A two‐stage approach was leveraged to extract the airway tree and trace the centerline of the tree structure.^13^ First, a CNN was trained to generate a probability map that represents the tree centerline. The training process involves utilizing a ground truth probability map, a manually traced airway centerline that undergoes a distance transformation by Gaussian smoothing. The probability map assigns higher probabilities to points closer to the center of the bronchi. Second, a tracing method called Rivulet was employed to derive the centerline from the generated probability map.^14^ This method utilizes iterative backtracking, which effectively handles small gaps that would otherwise result in disconnected segments. Through this combination of image‐to‐image probability map learning and iterative tracing, the complete airway tree was extracted.

#### 2.2.3 Segmental bronchi labeling

**FIGURE 3:**
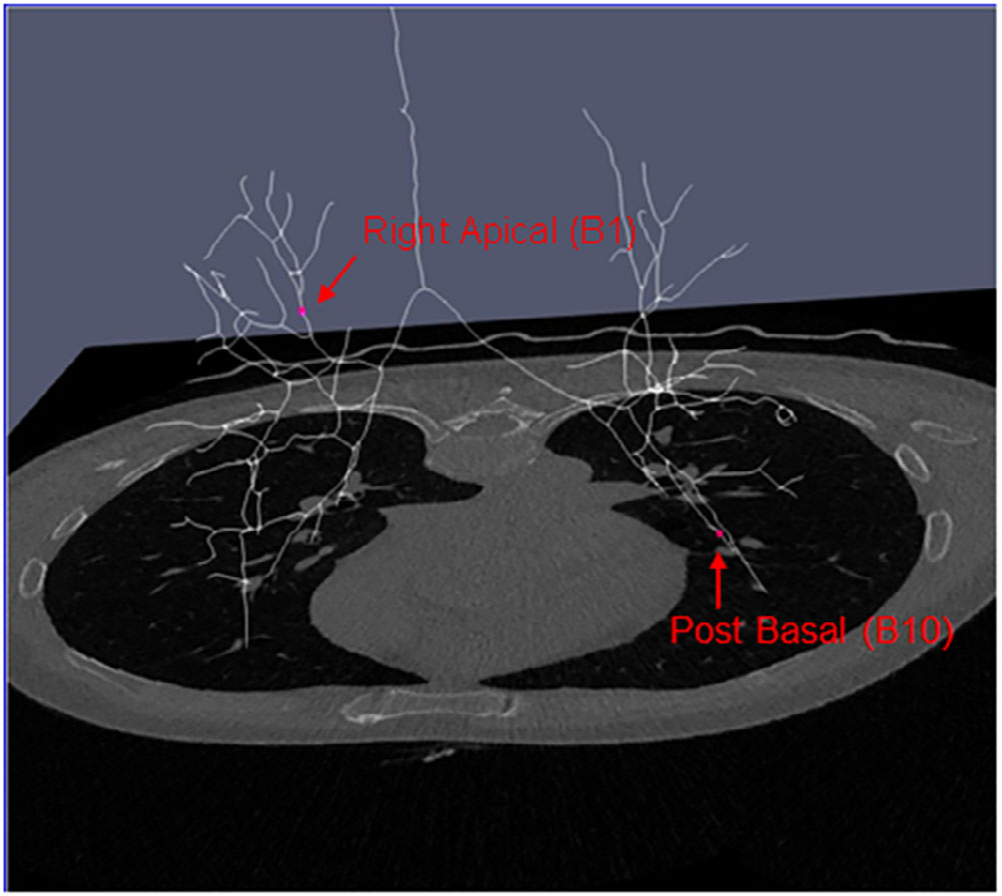
Segmental bronchi labeling. The extracted airway tree is loaded along with the corresponding CT image. Each time a segmental bronchus is identified, the annotator clicks on one point at the centerline and assigns a unique label to represent the specific segmental bronchus. CT, computed tomography.

We assigned labels to each segmental bronchus within the extracted airway tree. These segmental or tertiary bronchi, located at the third level of the airway tree (with the first level corresponding to the left and right main bronchi), serve as crucial anatomical references. Using the open‐source tool, *ParaView*, we loaded both the extracted airway tree and the corresponding image to provide the necessary anatomical context (Figure 3).^15^ The annotation task involved identifying and assigning a unique label to each of the 18 segmental bronchi (Table 1).

**TABLE 1:** Three levels of lung anatomy.

Right lung		Left lung	
Right upper lobe	Apical segment (B1)	Left upper lobe	Apicoposterior segment (B1/2)
	Posterior segment (B2)		Anterior segment (B3)
	Anterior segment (B3)		Superior lingular segment (B4)
Right middle lobe	Lateral segment (B4)		Inferior lingular segment (B5)
	Medial segment (B5)	Left lower lobe	Superior segment (B6)
Right lower lobe	Superior segment (B6)		Anteromedial basal segment (B7/8)
	Medial basel segment (B7)		Lateral basal segment (B9)
	Anterior basel segment (B8)		Posterior basel segment (B10)
	Lateral basel segment (B9)		
	Posterior basel segment (B10)		

Leveraging the hierarchical tree structure of the airways, the annotation process was simplified, with annotation only required at a single point within the segmental bronchus. This label was then propagated throughout the entire branch of that segmental bronchus and all its downstream sub‐branches, significantly reducing the manual effort required, allowing annotators to focus on accurately identifying the correct bronchi.

#### 2.2.4 Bronchi‐based parcellation

To generate a pulmonary segment mask, we leveraged both the lung lobe parcellation and the labeled segmental bronchi. For each voxel, *v*, within a given lobe, the parcellation process assigns the label of the segment corresponding to the segmental bronchus within the given lobe with the shortest distance, s(v), to *v*, that is, $s\;\left(v\right)=\underset{k}{\mathrm{argmin}}d_{k}\left(v\right)$, where $d_{k}\left(\cdot \right)$ represents the Euclidean distance from a voxel to the segment bronchi k. Background voxels from the lung lobes are also labeled as background for the pulmonary segments. The parcellation procedure benefits from the hierarchical relationship between lobes and segments. As a result, the search space for determining the appropriate segment label based on spatial proximity is limited to a maximum of five possibilities. Final segment masks were reviewed visually.

### 2.3 Development of parcellation algorithm and validation

Having established pulmonary parcellation, we proceed to train a deep image‐to‐image (DI2I) network to learn the mapping between images and corresponding labels. Starting from an end‐to‐end deep learning based automatic lung lobe parcellation algorithm, we incorporated an additional 123 thoracic CT datasets, with image quality commensurate with PET/SPECT attenuation correction, for segmental bronchi annotation purposes.^12^, ^16^ One hundred and fifteen were used for training and 8 for validation (for model selection). These datasets were annotated by two specialists with medical training following the steps described in section 2.1 above. Twenty independent test datasets were also annotated. The data were from multiple sources, with half referring to COPD and the rest with unknown clinical indication (but not specifically referring to lung disease).

**FIGURE 4:**
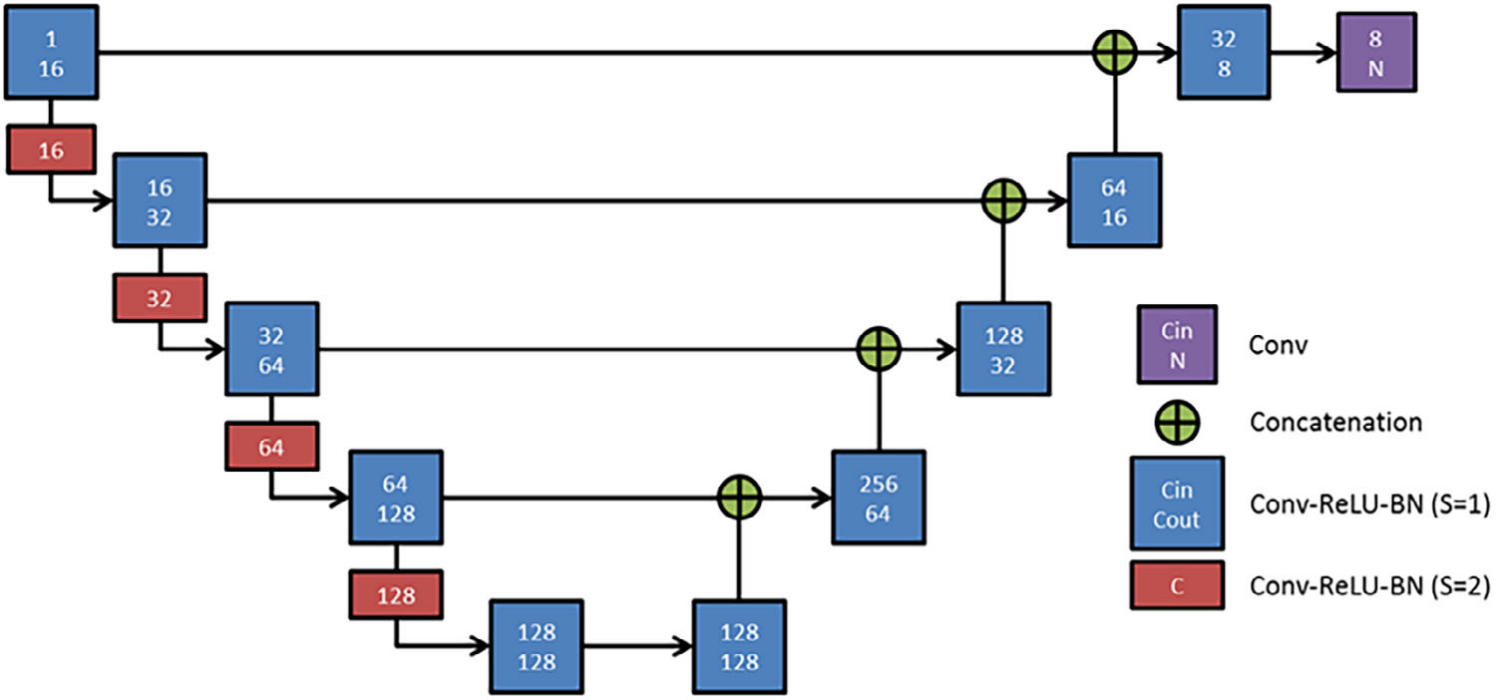
Network architecture. Deep Image‐to‐Image network architecture for organ parcellation (S: stride, Conv: convolution, ReLU: rectified linear unit, BN: batch normalization, N: number of output channels, N is set to 1 for single‐organ parcellation, and to 1 + number for organs.

The network architecture employed has been described previously.^17^, ^18^ This network's architecture comprises a convolutional encoder‐decoder structure with multi‐level feature concatenation (Figure 4).^17^ To guide the training process, we used the Lovasz loss function and the ADAM optimizer with a learning rate of 0.001.^19^, ^20^ During the inference phase, we leveraged deep reinforcement learning techniques to identify two landmarks, namely the carina bifurcation and sternum tips.^18^ These landmarks were used to crop a region of interest that encompasses both lungs, which is then resampled to 2mm isotopic and fed into the trained DI2I network for parcellation. To ensure robust segment parcellation, we incorporated the lung lobe parcellation to regularize the segment parcellation result, using a similar process to the one described in section 2.5 above.

The performance of the parcellation network was evaluated quantitatively using expert‐defined reference masks on twenty naive datasets, which were not used for training. The Dice scores 95% Hausdorff distance, and average symmetric surface distance (ASSD) between the manual parcellation and automatic parcellation results were calculated for each lung segment. A novel metric was also introduced, referred to here as the “inclusion rate”, which refers to the percentage of the detected bronchi in the airway traces covered by the correct segment.

We then sought to conduct qualitative external validation among a separate cohort of healthy adults (*n* = 10) and adults with COPD (*n* = 10) recruited at a large academic center with an aim of validating derived segments among those with and without architectural lung disease. Qualitative evaluations of the proposed lung segment parcellation algorithm were performed by two radiologists. Each reader scored each of the 18 bronchopulmonary segments as either a good match (e.g., generated segments do not cross associated bronchioles or fissures) or major mismatch (e.g., generated segments cross associated bronchioles or fissures).

## 3 RESULTS

### 3.1 Quantitative evaluation

Twenty CT images, including publicly available cases from the Open‐Source Imaging Consortium (OSIC), including health control and individuals with COPD (*n* = 5) were included for the creating of expert‐defined reference masks. Means and standard deviation of Dice score, 95% Hausdorff distance, ASSD, and inclusion rate between automatic and manual parcellation of 20 patient images were 86.81 (SD = 24.54), 26.97 (SD = 18.24), 5.38 (SD = 5.41) and 0.75 (SD = 0.19), respectively, across all lung segments.

### 3.2 Qualitative evaluation

Twenty adults with and without COPD were then recruited at a large academic center for external validation. The mean age was 54.4 years (SD = 16.4 years), with 45% (*n* = 9) female. Individuals with COPD had lower lung function (mean 51.6% predicted (SD = 30.0)) compared to the healthy group (mean 94.7% predicted (SD = 14.1), *p* = 0.005). There was an equal distribution of COPD by moderate and severe disease severity.

**FIGURE 5:**
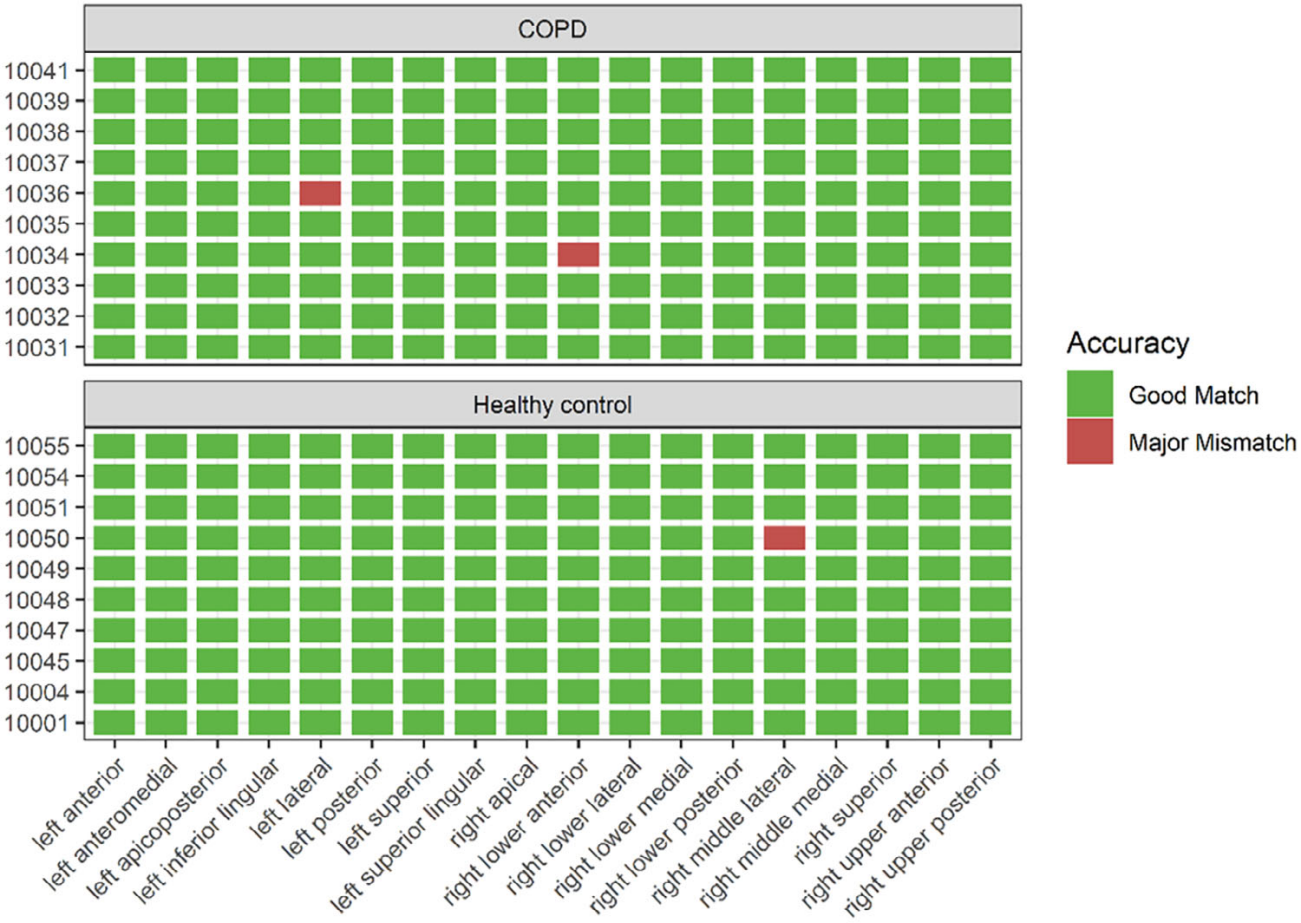
Qualitative evaluation of parcellation accuracy at a segmental level. Evaluation on parcellation masks on 10 healthy and 10 COPD subjects. A major mismatch was defined by the parcellation boundary erroneously crossing a bronchiole.

A total of 360 segments were analyzed by each radiologist. The percentage of segments that had a good match were 99.2% (357/360) (Figure 5). Among the major mismatch categories, the majority of major mismatches occurred among those with COPD (2/3). No specific segment had higher levels of mismatch.

**FIGURE 6:**
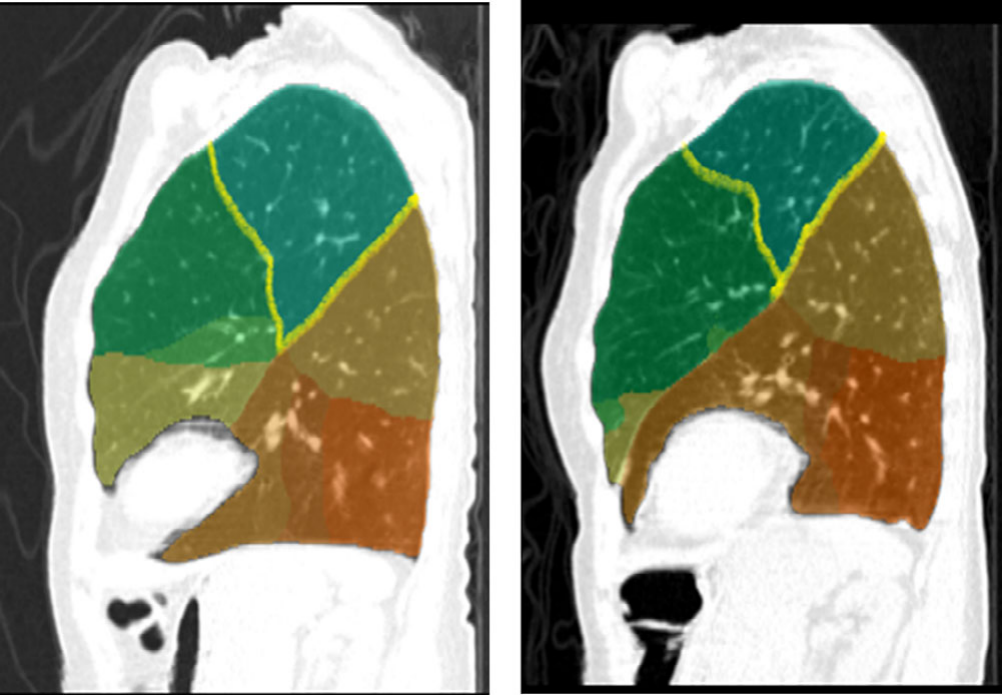
Lung segmentation before (left) and after (right) lung volume reduction. Yellow boundaries represent change in lung segmentation boundaries after placement of endobronchial valve.

We additionally demonstrate parcellation accuracy in the setting of lung volume reduction among a participant undergoing endobronchial valve placement. Despite a loss of volume in the target lobe, parcellation accuracy was preserved in all segments assessed (Figure 6).

## 4 DISCUSSION

In this study, which aimed to validate a deep learning CT‐based parcellation algorithm, we demonstrated high levels of agreement between the proposed parcellation model and trained radiologists in a clinical cohort of patients with and without airway disease. We utilized airway trees from high‐quality images and performed minimal manual effort (as compared to delineating the segmental structures slice‐by‐slice) to label the segmental bronchi. We then divided the entire lung into 18 sub‐structures of lung segments based on their spatial relationship to the labeled bronchi. Following this, an end‐to‐end parcellation framework was built upon a CNN to perform pulmonary segment parcellation from CT images. The validation of deep learning algorithms in a clinical cohort presents a necessary first step to wider adoption in clinical practice.

Several approaches were considered to automatically identify lung segments. We chose to adopt a CNN‐based approach because it has the capability to learn anatomical context extending beyond the airway bronchi. We also leveraged a previously developed CNN‐based lung lobe parcellation algorithm, which was trained on a very large dataset. Without being affected by the image quality, the lung segment parcellation neural network demonstrates the ability to learn the appropriate distribution of pulmonary segments by considering both local and global image features.

Airway‐based approaches can be negatively affected by the resolution, contrast enhancement, and overall quality of CT images. There is no guarantee of capturing up to the third level of pulmonary bronchi to support segmental parcellation. If a certain segmental bronchus is missed, it would nullify the derivation of the corresponding pulmonary segment. Given these uncertainties, robustly automating the bronchi identification and thus the segment parcellation becomes a challenging task.

Atlas‐based techniques are commonly used for aligning anatomies across subjects via registration. Multi‐atlas label fusion can potentially provide reasonable parcellation with only a small number of well‐annotated samples.^21^ This has been successfully applied to the human brain. However, to the best of our knowledge, such techniques have not been well adapted to pulmonary segments.^21^ The complexity and variations in shape and topology make it difficult to align pulmonary segments between subjects, even by using segmented lungs and extracted airways as supporting features on top of image registration.

Deep learning techniques, such as CNNs, have shown great potential in medical image parcellation, provided that a sufficient number of data pairs of input images and output parcellations are available. However, generating such data pairs can be cumbersome, particularly when attempting to obtain well‐curated pulmonary segments as ground truth. Each of the techniques described above has its own advantages, but none of them can be directly applied to address the challenges associated with pulmonary segment parcellation due to their inherent limitations.

There are broad clinical applications for lung parcellation in clinical practice. The present study was limited to patients with known obstructive airway disease, though the annotation framework can be integrated into several clinical scenarios. For lung resection, segment isolation can assist with decreasing the size of resection and improved prognostication of respiratory status post‐surgery. Furthermore, inclusion of SPECT or PET imaging can allow for overlay of the relative contribution of ventilation and perfusion per segment. Among individuals with COPD, ventilation heterogeneity has been well described. Identification of heterogeneity of ventilation and perfusion at a segmental level using the following deep learning framework can allow for early detection of disease as well as response to novel therapeutics targeting lung volume reduction and endobronchial valve placement as demonstrated in Figure 5. Furthermore recent approvals of biologic therapy pave the way for assessment of airway tree remodeling and improved ventilation across the zones of the lung.

There are several strengths to the present study, including a large derivation dataset and a separate validation cohort. Additionally, qualitative analysis was conducted prospectively on individuals with no respiratory disease and on those with COPD. There are several limitations noted, including the small validation cohort that may limit generalizability, as well as a disease specific focus on COPD. K‐fold validation could provide additional robustness to the model in future work. Although we found a high Dice score in the validation, the standard deviation of the Dice score demonstrated high variability, which is likely a reflection of the smaller sample size in this group. Further work is merited in other lung diseases with significant airway anatomy distortion (e.g., interstitial lung disease or lung cancer), where a higher rate of inaccurate parcellation may be encountered. Additional work is needed to compare the relative accuracy of lung parcellation modalities.

In summary, we (1) presented a feasible approach to assess lung segments from CT images on large scale datasets with reasonable manual efforts, (2) developed an automatic end‐to‐end workflow for lung segment parcellation, and (3) validated the proposed approach and demonstrated its efficacy both quantitatively on manually annotated data, as well as qualitatively on both healthy and COPD cohorts.

## AUTHOR CONTRIBUTIONS

Conceptualization: Trishul Siddharthan, Zhoubing Xu, Bruce Spottiswoode, Naresh Punjabi. Data Acquisition: Chris Schettino, Yoel Siegel, Michalis Georgiou, TE. Data Analysis: Trishul Siddharthan, Zhoubing Xu, Bernhard Geiger, Sasa Grbic. Interpretation of the Data: Trishul Siddharthan, Zhoubing Xu, Bruce Spottiswoode, Chris Schettino, Yoel Siegel, Michalis Georgiou, TE, BG, Sasa Grbic, Partha Gosh. Rachid Fahmi, Naresh Punjabi. Writing initial draft: Trishul Siddharthan, Zhoubing Xu, Bruce Spottiswoode, Naresh Punjabi. Critical review and editing of final manuscript: Trishul Siddharthan, Zhoubing Xu, Bruce Spottiswoode, Chris Schettino, Yoel Siegel, Michalis Georgiou, Thomas Eluvathingal, Bernhard Geiger, Sasa Grbic, Partha Gosh, Rachid Fahmi, Naresh Punjabi.

## CONFLICT OF INTEREST STATEMENT

Bruce Spottiswoode, Bernhard Geiger, Sasa Grbic, Partha Gosh, and Rachid Fahmi report employment at Siemens.

## ETHICAL APPROVAL

IRB approval obtained from the University of Miami (IRB 20210065).
